# The Influence of Chlorpyrifos on the Nonenzymatic Antioxidants Content in Macrophytes Leaves

**DOI:** 10.3390/antiox11040684

**Published:** 2022-03-31

**Authors:** Elżbieta Sobiecka, Milena Mroczkowska, Tomasz P. Olejnik

**Affiliations:** 1 Institute of Natural Products and Cosmetics, Faculty of Biotechnology and Food Sciences, Lodz University of Technology, ul. Stefanowskiego 2/22, 90-357 Lodz, Poland; milena.mroczkowska.500@guest.p.lodz.pl; 2 Department of Sugar and Food Safety Management, Lodz University of Technology, ul. Wolczanska 171/173, 90-924 Lodz, Poland; tomasz.olejnik@p.lodz.pl

**Keywords:** chlorpyrifos, phytoremediation, macrophytes, nonenzymatic defense system

## Abstract

Water pollution can be moved or limited by macrophytes in a phytoremediation process. The presence of insecticides in the plant life environment may affect physiological processes and contribute to the formation of secondary oxidative stress in plant tissues. To protect against oxidative stress, macrophytes have developed a system of mechanisms consisting of nonenzymatic and enzymatic antioxidants. The influence of various concentrations of chlorpyrifos on the nonenzymatic system in Canadian waterweed (*Elodea canadensis* Michx.), needle spikerush (*Eleocharis acicularis*) and water mint (*Mentha aquatica* L.) was studied. The differences in the value of the total concentration of polyphenols and flavonoids, as well as analysis of chlorophyll-a, chlorophyll-b, anthocyanin and carotenoid concentrations were determined in leaves. Research indicated a significant increase in the content of polyphenols and flavonoids in a solution with the highest concentration of chlorpyrifos while the opposite tendency was observed after analyses of the main assimilating pigments of plant tissues. It was concluded that aqueous plants exposed to toxic insecticide molecules created a defensive mechanism by nonenzymatic antioxidant systems and the amount of low-molecular weight compounds depended on the pollutant concentration which influenced biosynthesis mechanisms in plant cells.

## 1. Introduction

The intensive use of pesticides in agriculture can lead to significant threats to the functions of organisms living in neighboring aquatic and terrestrial ecosystems. The presence of plant protection products is found in all types of flowing water-precipitation, surface water and groundwater. In 2019, the Main Statistic Office of Poland (GUS), published the total sale of plant protection products which was estimated at 68,907 t. Herbicides accounted for 36,185 t. The next place was taken by fungicides at 17,858 t and insecticides at 8267 t [1]. The highest level of pesticides was found in the period of melt or floodwater runoff and during agrochemical treatments [2]. Some of the plants were used in the phytoremediation process to move toxic compounds from the environment. Even though the phytoremediation process was effective, the plants during growth and development were exposed to a variety of stress factors [3]. 

In cases of environmental stress, one of the first defensive reactions observed at the cellular level was the increase of reactive oxygen species concentration (ROS). This state generated damage to biologically important particles [4]. To protect their own cells from the harmful effects of high reactive oxygen species concentrations, the plants developed an efficient antioxidative system consisting of enzymatic and nonenzymatic antioxidants [5]. The nonenzymatic antioxidant system included, among others, phenolic compounds, flavonoids, pigments, such as chlorophyll-a and b, anthocyanins, carotenoids [6,7,8].

The results of our studies focused on the nonenzymatic antioxidative defense system (phenolic compounds, flavonoids, pigments, such as chlorophyll-a and b, anthocyanins, carotenoids) and changes caused by chlorpyrifos under abiotic stress [6,7,8]. It has been formed by low-molecular compounds which, by reacting with free-radical molecules, inhibited the reaction by “extinguishing” the excited oxygen molecules and dismutating peroxide radicals.

The literature review indicated that there is little information available on the effects of excessive concentrations of chlorpyrifos on compounds with antioxidant potential. Chlorpyrifos belongs to the organophosphorus insecticides group and was introduced to the market by the Dow Chemical Company in 1965 as a foliar pesticide [9,10]. It was also used to combat mosquitoes and other harmful insects in domestic and farm rooms. 

The synthesis of phenolic compounds in plants occurs under the influence of various external factors, such as pesticides. 

The antioxidant properties are a very valuable function of phenolic compounds; therefore, they are classified as natural antioxidant compounds [6,7,8]. The antioxidant properties of these polyphenols involve the elimination of reactive oxygen species, inactivation of free radicals, inhibition of enzymes from the oxidases group, as well as supporting enzymes showing antioxidant properties and chelation of metal ions (iron, copper) [11]. Thus, they protect the plant organism against oxidative stress [8]. Flavonoids, being derivatives of simple phenols, constitute one of the largest groups of plant secondary metabolites. Moreover, flavonoids are considered to determine the color of plants, as well as the smell and taste of fruits and flowers [12,13]. Not only do they perform important functions in the interaction between the plants and the external environment, but they are also natural repellents to other organisms [14,15]. Additionally, pigments create an important part of the nonenzymatic antioxidant defense system. Yet, the polluted environment causes changes in the concentrations of these compounds as an effect of abiotic stress. 

In this study, the total concentration of polyphenols, flavonoids and pigments (chlorophyll-a, chlorophyll-b, anthocyanins and carotenoids) were determined in selected macrophytes: Canadian waterweed (*Elodea canadensis* Michx.), needle spikerush (*Eleocharis acicularis*) and water mint (*Mentha aquatica* L.), which participated in the process of phytoextraction. 

The aim of our studies was to determine the total concentration of polyphenols, flavonoids and pigments (chlorophyll-a, chlorophyll-b, anthocyanins and carotenoids) in the selected macrophytes: Canadian waterweed (*Elodea canadensis* Michx.), needle spikerush (*Eleocharis acicularis*) and water mint (*Mentha aquatica* L.) in cultures without an insecticide and with various concentrations of chlorpyrifos. Plants could clean the surrounding environment in phytoremediation processes, but the toxic compound affected the plant’s physiology. 

## 2. Materials and Methods

### 2.1. Plants

Three species of plants naturally occurring in the water environment of our temperate climate in Poland were used in the research. The selected macrophytes came from organic farming: Canadian waterweed (*Elodea canadensis* Michx.), water mint (*Mentha aquatica* L.), needle spikerush (*Eleocharis acicularis*).

Three popular macrophytes for a temperate climate were investigated in our studies. The species of Canadian waterweed (*Elodea canadensis* Michx.), water mint (*Mentha aquatica* L.) and needle spikerush (*Eleocharis acicularis*) originated from organic farming. The experiment was conducted in aquariums to optimize the process parameters.

In the phytoremediation process, three selected plant species were cultivated. Then, the cleaned plants were planted in aquariums on aquarium gravel in an aqueous solution enriched with mineral salts: CaCl_2_·2H_2_O 0.106 g/dm^3^, MgSO_4_·7H_2_O 0.0038 g/dm^3^, Na_2_HPO_4_·12 H_2_O 0.0035 g/dm^3^, KH_2_PO_4_ 0.0138 g/dm^3^, Ca(HCO_3_)_2_ 0.272 g/dm^3^, FeSO_4_∙7H_2_O 0.0589 g/dm^3^, KCl 0.0038 g/dm^3^, NaNO_3_ 0.022 g/dm^3^ in a temperature of 23 ± 2 °C for 21 days in aerobic condition. The experimental set-up was provided in an aquarium with a day/night system at 21 ± 2 °C and a photoperiod of 14 h. The air humidity was measured by hydrometer (WSZ, Cracow, Poland). It was kept at 45–50% at a distance of 20 cm from the water surface. 

The solution was contaminated with different concentrations of chlorpyrifos: 50 μg/dm^3^, 100 μg/dm^3^, 150 μg/dm^3^. It is worth mentioning that macrophytes were also cultivated in the medium without adding the tested insecticide as a reference test. 

### 2.2. Determination of Polyphenols

The total polyphenols content was estimated by the Folin–Ciocalteu method as described by Singleton et al. with slight modifications [13]. The plant tissues were homogenized (1:3 *w*/*v*) with 80% methanol in a MASTICATOR Basic lab blender (IUL, Barcelona, Spain). The homogenate was centrifuged for 15 min at 20,000 rpm in a centrifugal separator of Sigma type 2-16P (Polygen, PL, Edgewood, NY, USA). The supernatant was used to measure polyphenol activity. 

The total polyphenols content was estimated by a method described by Singleton et al. with slight modifications [16]. The plant extract (0.250 mL) was mixed with 1.825 mL of distilled water and 50 µL of Folin reagent (Folin & Ciocalteu’s phenol reagent, Product Number: F9252, Sigma-Aldrich, St. Louis, MO, USA). After 3 min of incubation, 0.5 mL of sodium carbonate (10%) was added to the mixture to obtain an alkaline reaction environment. After 30 min of darkness, the absorbance was measured at λ 725 nm using a UV-Vis spectrophotometer UV/VIS 8453 Spectroquant Nova 400 (Merck KGaA, Darmstadt, Germany) with optical glass vials (Supelco 1.14946, rectangular cells 10 mm). The total content of polyphenols was determined as mg of gallic acid equivalents per gram of plant fresh weight (mg GAE. g^−1^ f.w.). 

### 2.3. Determination of Flavonoids Content

The flavonoids content was based on the Lamaison and Carnat procedure with slight modifications. The plant tissues were homogenized (1:3 *w*/*v*) with 80% methanol in a MASTICATOR Basic lab blender (IUL, Barcelona, Spain). The homogenate was centrifuged for 15 min at 20,000 rpm in a centrifugal separator of Sigma type 2-16P (Polygen, PL). 

The colorimetric method with aluminum chloride (AlCl_3_) was used for flavonoid determination [17]. The 0.5 mL extract solution was mixed with 1.5 mL 80% methanol, 0.1 mL 10% AlCl_3_ and 0.1 mL 1 M sodium acetate solution. After 30 min of incubation in darkness, the mixture turned yellow and the absorbance was measured at λ 415 nm using a UV-Vis spectrophotometer UV/VIS 8453 Spectroquant Nova 400 (Merck KGaA, Darmstadt, Germany) with optical glass vials (Supelco 1.14946, rectangular cells 10 mm), against a blank solution containing the reaction mixture, in which the supernatant was substituted with 80% methanol. The total flavonoids content was expressed based on the calibration curve as mg of quercetin per 1 g of plant fresh weight (mg QE/g f.w.).

### 2.4. Determination of Pigments

The pigments: chlorophyll-a, chlorophyll-b, carotenoids and anthocyanins were determined by Hiscox and Israelstam method [18]. The sample of plant tissue (0.2 g) was mixed with 5 mL dimethyl sulfoxide (DMSO). Next, the samples were incubated in darkness at 65 °C in a shaking water bath GFL-1083 (Germany) for 30 min. The UV/VIS 8453 spectrophotometer Spectroquant Nova 400 (Merck KGaA, Darmstadt, Germany) with optical glass vials (Supelco 1.14946, rectangular cells 10 mm) was used to detect pigments at the wavelengths: chlorophyll-a at λ 661 nm, chlorophyll-b at λ 643 nm, carotenoids at λ 470 nm, anthocyanins at λ 534 nm. The content of plant pigments was calculated according to the Arnon formula [19]. The number of individual pigments was expressed as mg of plant fresh weight per 1 g (mg/g f.w.).

### 2.5. Statistical Analysis

The results obtained were analyzed statistically in STATISTICA Version 10. The presented results are the average of three independent biological repetitions. They were subjected to a single-factor analysis of ANOVA variance and then analyzed using Duncan’s multiple ranges posthoc test (*p* < 0.05) in order to show statistically significant differences between the tested samples.

## 3. Results

The studies focused on the analyses of selected antioxidants changes, metabolized by aquatic plants during oxidative stress caused by the presence of chlorpyrifos in water.

**Figure 1:**
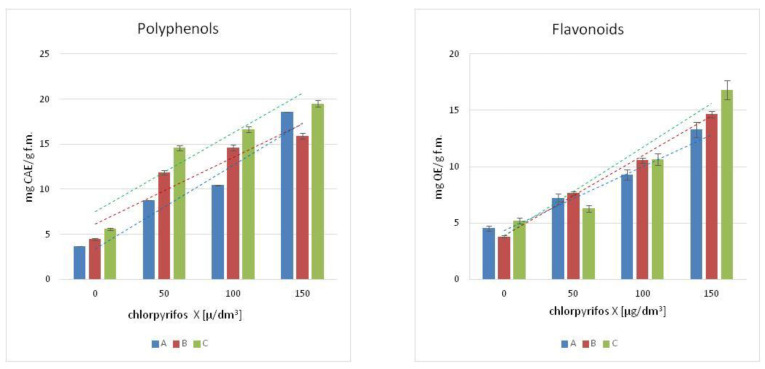
The content of: Polyphenols and Flavonoids in A-Canadian waterweed (*Elodea. canadensis* Michx.), B-needle spikerush (*Eleocharis acicularis*), C-water mint (*Mentha aquatica* L.). Linowa means linear curve.

The content of polyphenols was measured in the leaves of the chosen macrophytes, Figure 1. The increased concentration of chlorpyrifos in the cultures resulted in an increase of polyphenols in the tissues of the tested plants, and the highest concentration of phenolic compounds was observed in the culture treated with the highest concentration of pesticide (150 µg/dm^3^). The growth was more than 4-fold for Canadian waterweed and mint, respectively, for the control plants, and more than 3-fold for needle spikerush. A similar tendency was observed in flavonoids contents, Figure 1. The highest concentration of flavonoids was observed in the leaves of water mint exposed to the highest concentration of chlorpyrifos (150 µg/dm^3^).

As for the Canadian waterweed plants, both needle spikerush and mint, a higher content of flavonoids was observed in plants growing in a culture polluted by a 150 µg/dm^3^ concentration of chlorpyrifos. The highest flavonoids content was observed in water mint.

**Figure 2:**
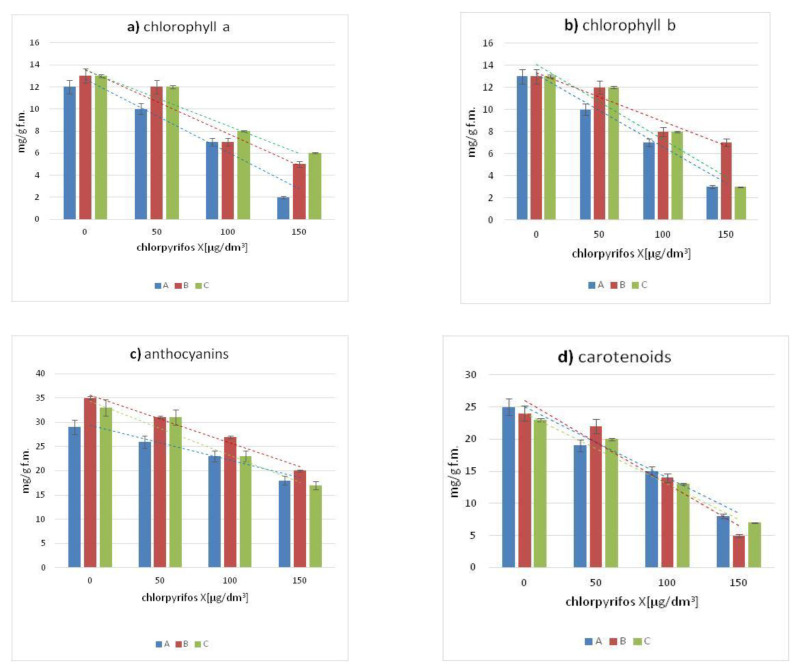
The content of: (**a**) chlorophyll-a; (**b**) chlorophyll-b; (**c**) anthocyanins; (**d**) carotenoids in A-Canadian waterweed (*Elodea canadensis Michx*.), B-needle spikerush (*Eleocharis acicularis*), C-water mint (*Mentha aquatica* L.). Liniowa means linear curve.

Figure 2 presents the average content of four pigments in the leaves of Canadian waterweed, needle spikerush and water mint. In comparison with the control cultures, in the leaves of all tested plants exposed to chlorpyriphos, the concentration of chlorophyll-a and b decreased with the increase of pesticide concentration. All the plants studied showed a decrease in concentrations of chlorophyll-a, chlorophyll-b, anthocyanins and carotenoids were observed in all plants in comparison with the plant cultures under control conditions. The most significant decrease in the content of the previously mentioned pigments compared to the control sample was observed in leaves growing in an environment contaminated by the highest concentration of chlorpyrifos (150 µg/dm^3^) as well as all tested pesticide concentrations.

The significant decreases in chlorophyll-a and b content as compared to control plants were observed for Canadian waterweed and water mint growing in the environment containing the highest concentration of chlorpyrifos (150 µg/dm^3^).

A synchronous decrease in the content of anthocyanins and carotenoids was also observed for the examined plants in the cultures conducted, along with an increase in the pesticide concentration. The highest content of the above-mentioned pigments was characteristic for control plants, while the lowest value was shown by all plants growing in an aquatic environment contaminated with the highest concentration of chlorpyrifos.

The presented results of the studies were an effect of the phytoremediation process. Three selected macrophytes species were cultivated in lab conditions, in aquariums on aquarium gravel in an aqueous solution enriched with mineral salts for 21 days. After that time, in plants exposed to high concentrations of chlorpyrifos, (100 μg/dm^3^ and 150 μg/dm^3^) necroses of leaves, root systems and even entire plants were observed. This made it impossible to conduct any further experiments.

The effect of the chlorpyrifos concentration in the tested plants changed the content of polyphenols, flavonoids and pigments concentrations. The obtained results suggested that these changes can be calculated by a linear function. The model has been described with the equations presented in Table 1. The high value of the correlation coefficients R^2^ (Table 1) confirmed the proper use of the linear functions in the model description. Figure 1 and Figure 2 include the correlation curves as the graphical proof of the model calculations. The color of the curves corresponds to the colors of the columns in the individual diagrams for the plant species studied.

## 4. Discussion

The widespread usage of plant protection products, including organophosphorus insecticides, has caused changes in terrestrial and aquatic ecosystems for many years. Therefore, plants had to adapt to the presence of toxic compounds in the environment by developing defensive systems allowing them to live in new conditions.

Excessive concentrations of pesticides in the environment lead to increased uptake, accumulation in plant tissues and can contribute to inducing oxidative stress resulting from the overproduction of reactive oxygen species which are dangerous for the plant cell. Plants have developed a system of precisely working antioxidants to defend against oxidative damage. Some plant species are resistant to contamination and have the ability to accumulate and assimilate pollutants by building them into the structure of their own cells [20].

The literature review indicates that there is little information available on the effects of excessive concentrations of chlorpyriphos on compounds with antioxidant potential, phenolic compounds and flavonoids.

The highest concentration of phenolic compounds was observed in leaves in all examined plants growing in culture treated with the highest concentration of chlorpyrifos (150 µg/dm^3^). Phenolic compounds constitute a numerous and very diverse group of strong antioxidants, capable of “scavenging” free radicals. The relationship between the content of phenolic compounds and the total antioxidant potential suggests that phenolic compounds constitute the dominant group of antioxidants in the tissues of selected plants. They have strong antioxidant properties consisting of the elimination of RFT and inactivation of free radicals by binding harmful substances and the protection of plant tissues against oxidative stress caused by environmental pollution [18].

It can be concluded on this basis that plants have developed mechanisms to enable them to grow in environments contaminated by various types of pollution.

Toxic substances accumulate in the vacuole by producing complexes with phenol derivatives, which prevent them from moving to other plant tissues. These mechanisms detoxify the plant and lead to the removal of contaminants by active transport [21].

Due to their chemical structure, flavonoids showed strong antioxidant properties. Numerous experiments proved that they can be characterized by the inhibition of reactive oxygen species (ROS) formation, chelation, reduction of transition metal ions, ROS capture, and interruption of the free radical reaction cascade (through lipid and alkoxyl radical traps leading to lipid peroxidation) [22].

The highest concentration of flavonoids was observed in the leaves of water mint growing in assisted cultures exposed to the highest concentration of chlorpyrifos (150 µg/dm^3^).

In the presence of toxic compounds, such as pesticides, the plants activated the cells signaling processes that can result in transcriptional up-regulation of the phenylpropanoid pathway [23]. This stimulated phenolic biosynthesis by activating the proper biosynthetic enzymes and up-regulation of key genes of the phenylpropanoid branch, including phenylalanine ammonia lyase and chalcone synthase [23,24,25].

Plants subjected to difficult conditions in chlorpyrifos contaminated cultures may show accelerated aging. The negative effect of chlorpyrifos on the content of chloroplast pigments in the leaves of the tested plants may result in oxidative stress through increased production of free radicals causing peroxidation of chloroplast membranes and inhibition of the activity of enzymes involved in the process of chlorophyll synthesis [26].

The pigment changes in leaves can affect the ability of plants to grow and develop the ability to perform photosynthesis. Capturing light energy has been the primary function of photosynthesis. The first stage is the absorption of light by the photoreceptor molecule, which in green plants is been chlorophyll-a. The decrease of chlorophyll-a and other substances supporting it in the process of biochemical transformations affect changes in the proper photosynthesis mechanism. Other pigments, such as chlorophyll-b or carotenoids, direct light energy to reaction centers where it is converted into chemical energy to be used by plants cells [27].

All the plants studied showed a decrease in chlorophyll-a, chlorophyll-b, carotenoids and anthocyanins as compared to growing species under control conditions. The greatest decrease in the content of the previously mentioned dyes compared to the control sample was observed in leaves growing in an environment contaminated by the highest concentration of chlorpyrifos (150 µg/dm^3^), as well as all tested pesticide concentrations.

The decrease of chloroplast pigments might be the result of a general inhibition of the growth and development of these plants (leaf withering and dying) growing in an environment exposed to chlorpyrifos. 

Similar results were obtained by the Shixiang group carried out on the hornwort (*Ceratophyllum demersum*), the eelgrass (*Vallisneria natans*) and Nutall’s waterweed (*Elodea nuttallii*) growing under exposure to different concentrations of butachlor, bensulfuron-methyl and atrazine (0.0001 mg/L; 0.005 mg/L; 0.05 mg/L and 0.5 mg/L) in a culture lasting 21 days [28]. The concentration of chlorophyll-a decreased as the concentration of pesticides increased. The highest decrease in pigment content in the studied plants was observed as a result of exposure to the highest concentrations of butachlor, bensulfuron-methyl and atrazine. A positive correlation between decreasing chlorophyll content and increasing herbicide concentrations means that pesticides have a negative effect on macrophyte patterns.

Yadav’s group observed a decrease in chlorophyll in *Spirulina platensis* after 6-day exposure to different concentrations of chlorpyrifos (0.5; 1.0; 5; 10 and 15.0 ppm). The most stimulating effect on pigment content was observed at the highest pesticide concentration (15 ppm). The content of the pigment studied was completely dependent on the concentration of chlorpyrifos because with the increase in the concentration of the pesticide a decrease in chlorophyll-a was observed [29].

The tested plants: Canadian waterweed (*Elodea canadensis* Michx.), water mint (*Mentha aquatica* L.) and needle spikerush (*Eleocharis acicularis*), created a defense system against oxidative stress caused by an organophosphorus insecticide. The nonenzymatic antioxidative system described above indicated that pollutants influenced the synthesis of low-molecular weight compounds. The decrease of four investigated pigments, as well as the increase of polyphenols and flavonoids, can be an effect of disruption of the natural mechanism in the cell organelles that appeared after chlorpyrifos contamination.

## 5. Conclusions

Three species of macrophytes: Canadian waterweed (*Elodea canadensis* Michx.), water mint (*Mentha aquatica* L.) and needle spikerush (*Eleocharis acicularis*), that naturally have existed for a temperate climate were investigated. The water environment was polluted by chlorpyrifos, one of the most used and popular insecticides. The toxic compound influenced four groups of nonenzymatic compounds: polyphenols, flavonoids and pigments (chlorophyll-a, chlorophyll-b, anthocyanins and carotenoids) being the key elements of the nonenzymatic antioxidative stress system. The content of the low molecules depended on chlorpyrifos concentrations.

Based on the analysis of the results obtained, it is concluded that aqueous plants exposed to toxic insecticide molecules created a defensive mechanism by nonenzymatic antioxidant systems.

Contamination of the aquatic environment with 150 μg/dm^3^ chlorpyrifos resulted in an increase of polyphenols and flavonoids in plant leaves while the content of pigments (chlorophyll-a, chlorophyll-b, anthocyanins and carotenoids) decreased. The observed effect was directly connected with biosynthesis mechanisms conducted in plant cells.

## Figures and Tables

**Table 1:** The equations and correlation coefficients for the model describing changes in pigments concentration.

Nonenzymatic Antioxidant	Plant	Equation	R^2^
Polyphenols	A	f_A_(x) = 4.639 x −1.225	0.9378
	B	f_B_ (x) = 3.708 x + 2.430	0.8745
	C	f_C_(x) = 4.378 x + 3.14	0.8856
Flavonoids	A	f_A_(x) = 2.825 x + 1.535	0.9815
	B	f_B_(x) = 3.548 x + 0.305	0.9960
	C	f_C_(x) = 3.916 x − 0.050	0.9200
Chlorophyll-a	A	f_A_(x) = −3.3 x + 16.0	0.9595
	B	f_B_(x) = −2.9 x + 16.5	0.9397
	C	f_C_(x) = −2.5 x + 16,0	0.9542
Chlorophyll-b	A	f_A_(x) = −3.3 x + 16.5	0.9945
	B	f_B_(x) = −2.2 x + 15.5	0.9308
	C	f_C_(x) = −3.4 x + 17.5	0.9323
Anthocyanins	A	f_A_(x) = −3.6 x + 33.0	0.9818
	B	f_B_(x) = −4.9 x + 40.5	0.9780
	C	f_C_(x) = −5.6 x + 40.0	0.9561
Carotenoids	A	f_A_(x) = −5.5 x + 30.5	0.9902
	B	f_B_(x) = −6.5 x + 32.5	0.9399
	C	f_C_(x) = −5.5 x + 29.5	0.9774

**A**-Canadian waterweed (*Elodea canadensis* Michx.), **B**-needle spikerush (*Eleocharis acicularis*), **C**-water mint (*Mentha aquatica* L.).

## Data Availability

The data is contained within the article.
